# Integrative analysis reveals CRHBP inhibits renal cell carcinoma progression by regulating inflammation and apoptosis

**DOI:** 10.1038/s41417-019-0138-2

**Published:** 2019-10-01

**Authors:** Kang Yang, Yusha Xiao, Tao Xu, Weimin Yu, Yuan Ruan, Pengcheng Luo, Fan Cheng

**Affiliations:** 1 Department of Urology, Renmin Hospital of Wuhan University, Wuhan, Hubei China; 2 Department of General Surgery, Research Center of Digestive Diseases, Zhongnan Hospital of Wuhan University, Wuhan, Hubei China; 3 Central Hospital of Edong Healthcare Group, Hubei Key Laboratory of Kidney Disease Pathogenesis and Intervention, Hubei Polytechnic University, Huangshi, China

**Keywords:** Gene regulation, Renal cancer

## Abstract

Patients with renal cell carcinoma (RCC) usually develop drug resistance and have poor prognosis owing to its insensitive property. However, the underlying mechanisms of RCC are still unclear. We implemented an integrative analysis of The Cancer Genome Atlas and Gene Expression Omnibus datasets. Three genes (CRHBP, RAB25 and PSAT1) were found to be potential biomarkers in ccRCC and validated by four independent cohorts. Then, ccRCC patients with a decreased expression of CRHBP in tumor tissues had significantly poor survival by TCGA ccRCC datasets and verified by clinical samples as well as RCC cell lines. Overexpression of CRHBP suppressed cell proliferation, migration, invasion as well as apoptosis in vitro and in vivo. Moreover, the results of western blot analysis showed the effects of CRHBP via upregulating NF-κB and p53-mediated mitochondria apoptotic pathway. Our results suggested that CRHBP may be an effective target to treat ccRCC patients.

## Introduction

Renal cell carcinoma (RCC), the third most common malignancy of the urinary system, is the sixth most frequently diagnosed malignant neoplasms in men and tenth in women accounting for 4% of all cancers [1, 2]. Clear renal cell carcinoma (ccRCC), one of the most frequently histologic subtypes of RCC, derives from the renal parenchyma urinary tubule epithelial cell representing ~70–85% of all RCC diagnoses [3]. Due to unconspicuous symptoms in early stage of ccRCCs, about 30% patients have localized or distant metastasis at diagnosis [4]. Current numerous research results concerning the mechanisms of ccRCC are based on the small sample, and the evidence of whole-genome sequencing only indicated some somatic mutant genes are linked with pathogenesis of ccRCC. Therefore, it is imperative to further explore and recognize the crucial biomarkers that help us uncover more comprehensive mechanisms and against the proliferation and progression of ccRCC.

In the last decade, great advancement of high-throughput sequencing technology enabled us to bring a comprehensive molecular insight on ccRCC [5, 6]. According to the next-generation sequencing technology, numerous differentially expressed genes (DEGs) have been served as biomarkers for ccRCC pathogenesis or potential therapeutic targets. Recent studies showed that corticotropin releasing factor binding protein (CRHBP) expression is linked with high α-fetoprotein level in patients with hepatocellular carcinoma (HCC), and HCC patients with low CRHBP expression have poor overall survival rate [7]. Moreover, hypermethylation level of CRHBP was significantly negative with mRNA expression based on methylated microarray, and alteration of methylation level could affect ccRCC cell lines migration and invasion [8]. However, the clinical practical value of these studies is still limited owing to lack of several independent cohorts to validate. Besides, few potential mechanistic researches reveal how CRHBP affects the progress and metastasis of ccRCC.

In our study, two popular microarray databases from The Cancer Genome Atlas (TCGA) and gene expression omnibus (GEO) are selected to integratively analyze and explore DEGs between ccRCC tumors and adjacent normal samples. We then performed survival analysis and regress analysis to define the relationship between ccRCC patients and important genes, and constructed a predictive model to assess the contribution of important genes that acted as suitable and valuable biomarkers for ccRCC. Three genes named *CRHBP*, *PSAT1* and *RAB25* were significantly linked with the progression of ccRCC by support vector machine recursive feature elimination (SVM-RFE) and least absolute shrinkage and selector operation (LASSO) regression model as well as cox regression model. Four independent ccRCC microarrays were utilized to validate the crucial genes. At the same time, these genes were validated by 24 pairs of clinical ccRCC samples as well as four ccRCC cell lines. CRHBP, the gene with the most significant variation in expression was selected to elucidate the underlying molecular mechanism in vitro and in vivo. We explored the influences of CRHBP on tumor cell migration and invasion. In addition, we demonstrated that CRHBP could affect inflammation and apoptosis of ccRCC by targeting NF-κB and p53-mediated mitochondria apoptotic pathway.

## Materials and methods

### Acquisition RNAseq data and clinical characteristics

The mRNA data expressions of GSE16449, GSE36895, GSE53757, GSE66270, GSE68417, GSE66272 and GSE76351, as well as other mRNA expression files (level 3), were downloaded from GEO (http://www.ncbi.nlm.nih.gov/geo/) database and TCGA database, respectively. Besides, a total of 539 ccRCC clinical informations were also obtained from TCGA database (https://tcga-data.nci.nih.gov/tcga/). After excluding patients without entire follow-up information, 491 patients with clinicopathological parameters including age, gender, pathological stage (according to the seventh edition AJCC) and neoplasm grade remained available for analysis.

### Data preprocessing and identification of DEGs

Briefly, all raw gene datasets in GEO were background corrected, normalized separately by Robust Multi-Array Average method [9], and annotated to gene symbol by GEO platforms. The microarray data of TCGA was annotated by the general feature format files based on alignments of biological evidence in ensemble database (http://asia.ensembl.org/index.html). After preprocessing, Linear Models for Microarray (Limma) package and empirical analysis of digital gene expression data in R (EdgeR) package [10] were used for identifying the DEGs between the ccRCC patients and the normal samples. Fold changes (FC) and adjusted *P*-value were individually calculated by the two packages as well as Benjamini–Hochberg method [2] in each expressed gene. The DEGs with an adjusted *P* < 0.05 and |log_2_FC| ≥2 were considered statistically significant.

### Functional analysis and construction of gene-related prognostic model

Database for annotation, visualization and integrated discovery (DAVID) (http://david.abcc.ncifcrf.gov/) was used for elucidating the biological properties such as biological process (BP), cellular component (CC) and molecular function (MF) of the intersecting DEGs. Moreover, pathway enrichment analysis of the DEGs was performed using gene set enrichment analysis (GSEA). LASSO cox regression model [11], a popular method with penalty parameters tuning determined by tenfold cross-validation for regression analysis with high dimensional predictors, was utilized to select important ccRCC associated genes as prognostic biomarkers by LASSO and elastic-net regularized generalized linear models (Glmnet) package in software R (version 3.5.2). Meanwhile, SVM-RFE [12, 13], another algorithm filtering out noise and non-informative variables by means of artificial variables and mutual information, was used for crucial genes. Furthermore, cox regression model with the other clinical characteristics was used to further assess and optimize the contribution of vital genes as independent prognostic variables for patient survival.

### Tissue collection

Forty-eight frozen tissue specimens included 24 tumor tissues and 24 corresponding non-tumor tissues were obtained from ccRCC patients who had been diagnosed with ccRCC by pathologists in Renmin Hospital of Wuhan University between April 2014 and January 2015. All acquired samples were collected immediately after surgical resection, and snap-frozen in liquid nitrogen and stored at −80 °C until RNA extraction. All participating patients signed informed consent and all protocol for this research was approved by the Research Ethics Committee of Renmin Hospital of Wuhan University.

### RCC cell lines and lentivirus infection

HK-2 derived from normal human kidney, and Caki-1,769-P, ACHN as well as 786-O human RCC cell lines were purchased from the Cell Bank of Wuhan University and the Cell Bank of Type Culture Collection (CBTCC, Chinese academy of sciences, Shanghai, China). Minimum essential medium and RPMI 1640 medium (Gibco, Carlsbad, CA, USA) supplemented with 10% fetal bovine serum (FBS) (Gibco, Carlsbad, CA, USA) and 1% streptomycin–penicillin (Gibco, Carlsbad, CA, USA) were, respectively, used for HK-2 and ACHN as well as Caki-1, 769-P and 786-O cell culture. All cell lines were maintained in 37 °C with 5% CO_2_ humidified environment. To construct stable overexpression of CRHBP, lentivirus containing pLVX-Puro (Vector) and pLVX-CRHBP-Puro obtained from Genechem (GeneChem, China) was used to infect 769P and ACHN according to the manufacturer’s protocol.

### RNA extraction and quantitative reverse transcription polymerase chain reaction (qRT-PCR)

Total RNA from RCC specimens and RCC cell lines were extracted using a Trizol reagent (Invitrogen, USA) and reverse transcribed to produce complementary DNA performing with RevertAid First Strand cDNA Synthesis Kit (Thermo, USA). qRT-PCR was performed using with SYBR Green Mix (Takara, Japan) in the CFX Connect Real-Time PCR Detection System (Bio-Rad, USA) according to the manufacturer’s instructions. The relative expression of *CRHBP* was calculated by 2^−ΔΔCt^ method and *GAPDH* expressed level served as an internal control to normalize the expression. The primers involved in our study are listed as follows:

CRHBP forward: 5′-TTCATTACCATCCACTACGA-3′;

CRHBP reverse: 5′-CTCCTGCTAAGACCACTC-3′;

GAPDH forward: 5′-CCTTCATTGACCTCAACTACA-3′;

GAPDH reverse: 5′-GCTCCTGGAAGATGGTGAT-3′.

### Western blotting

Briefly, total protein from tumor samples and cell lines was isolated using RIPA lysis buffer (Servicebio, China), and protein concentration was measured by BCA Protein assay kit (Thermo Fisher Scientific, UAS, 1:1000). Western blot analysis of CRHBP (Thermo Fisher Scientific, UAS, 1:1000), P-AKT (Cell Signaling Technology, USA, 1:1000), AKT (Cell Signaling Technology, USA, 1:1000), p-p65 (Cell Signaling Technology, USA, 1:1000), p65 (Cell Signaling Technology, USA, 1:1000), P-IκBα (Cell Signaling Technology, USA, 1:1000), IκBα (Cell Signaling Technology, USA, 1:1000), p-p53 (Cell Signaling Technology, USA, 1:1000), p53 (Cell Signaling Technology, USA, 1:1000), Bax (Cell Signaling Technology, USA, 1:1000), Bcl2 (Cell Signaling Technology, USA, 1:1000), Bcl-2 (Cell Signaling Technology, USA, 1:1000), Bcl-xl (Cell Signaling Technology, USA, 1:1000), Cytochrome c (Cell Signaling Technology, USA, 1:1000), β-actin (Abcam, Cambridge, UK, 1:5000) and GAPDH (Cell Signaling Technology, USA, 1:5000) was according to standard protocols. The blots were scanned with two-color infrared imaging system (Odyssey, LI-COR, USA) after incubating with goat-anti-rabbit secondary body (LI-COR, USA, 1:2000), and analyzed by image J software.

### Cell proliferation assay

Cell Counting Kit-8 (CCK8, Japan) was employed to test the proliferation rate based on the manufacturer’s protocols. Briefly, 769P and ACHN cells with CRHBP overexpression and vector ccRCC cells were plated in 96-well plates in triplicate at 10^4^ cells per well in 100 µl of culture medium. CCK8 solution was added into each well, and the plate was then incubated for 1 h at 37 °C. Finally, treated cells were measured at the absorbance of 450 nm at the indicated time points.

### Wound healing and transwell invasion assays

769-P and ACHN cell lines in each group were respectively plated into 6-well plates for 24–48 h, and vertical wound was created by 200 µl sterile pipette tip when the density of cell reached at a density of 90%. Then, the cells were cultured in serum-free media and the image of cell migration was obtained 0 and 24 h later by inverted microscope (Olympus, Japan). Matrigel chamber (BD Biosciences, USA) was used for validating the migration ability of cells. Briefly, 3 × 10^4^ cells with serum-free medium were added into each upper chamber and complete medium with 10% FBS was added into the corresponding lower chambers to provoke the cell invasion. After incubation for 24 h, the cells that have passed through the membrane and attached into the bottom surface were fixed and stained with 4% paraformaldehyde and 0.1% crystal violet solution. Then, the stained cells were observed and counted in five random fields under inverted microscope, and the results were analyzed by ImagePro Plus 5.0 (Media Cybernetics, MD).

### Immunofluorescence assay

Cells were fixed with 4% formaldehyde for 20 min and washed with PBS, then permeabilized with 0.1% Triton X-100 and blocked with 0.5% goat serum for 1 h at room temperature. Subsequently, cell lines were incubated with CRHBP (Thermo Fisher Scientific, UAS,1:100) and cleave caspase 3 (Cell Signaling Technology, USA, 1:100) antibodies for overnight, followed by Alexa Fluor 488 and 555 (Cell Signaling Technology, USA, 1:200) goat anti-rabbit secondary antibodies. The nuclei of the cells were stained with DAPI in dark, and images were captured by fluorescence microscope (Olympus, Japan), and the results were analyzed by ImagePro Plus 5.0 (Media Cybernetics, MD).

### Flow cytometry assay

769-P and ACHN cells were infected with lentivirus, and stably overexpressed CRHBP; then the cells were stained with Annexin V-FITC and propidium iodide (PI) and analyzed by flow cytometry based on the manufacturer’s instructions (BD Biosciences, USA).

### Tumor xenograft model

Five-week-old male BALB/c nude mice (20 ± 2 g) were obtained from the Animal Center of Wuhan University (Wuhan, China) and divided randomly into two group (*n* = 5 per group). A total of 200 µl of cell suspension of 769-P cells (2 × 10^6^) stably transfected with CRHBP lentivirus was subcutaneously injected into the left armpits of the mice. All mice were sacrificed after 28 days injection, and the xenografts were harvested for further assessment. The animal experiments involved in this study were approved by the Animal Care and Use Committee of Wuhan University Renmin Hospital.

### Statistical analysis

Two groups were compared using Student's *t* test for continuous variables and *χ*^2^ test for category variables. The Kaplan–Meier method was utilized to analyze the correlation between categorical variables and overall survival, and the log-rank test was used for comparing the survival curve of two groups. Cox’s proportional hazards regression model was constructed by univariate and multivariate analysis, and the variables with statistical significance in the univariate analysis were incorporated into the multivariate analysis. In the predictive model, each sample was assigned a risk score referring to the Cox regression coefficient of significant variables [14, 15], and receiver operating characteristic (ROC) curve estimated the sensitivity and specificity of survival prediction. All data for bioinformatic analysis in this study were processed by Software R (version 3.5.2, https://www.r-project.org/). All statistical analysis was performed with GraphPad Prism version 7.0 software (GraphPadSoftware, USA), and the results were represented as mean ± standard deviation (SD) at three independent experiments. Differences with *P* value < 0.05 were considered statistically significant.

## Results

### Integrates analysis of four ccRCC microarray datasets identified three significant genes in ccRCC

**Fig. 1:**
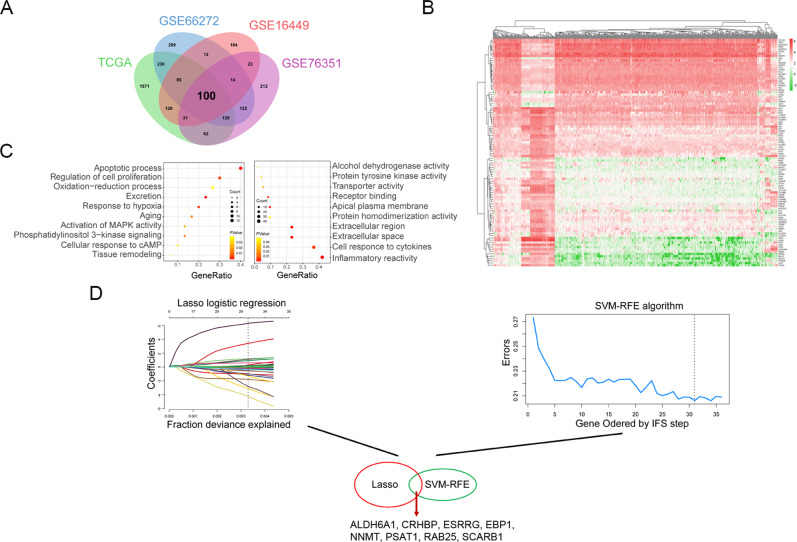
Identification of important differentially expressed genes (DEGs) for classifying normal and ccRCC patients. **a** Venn diagrams of TCGA, GSE66272, GSE16449 and GSE76351 datasets DEGs. **b** Clustering of the 100 DEGs between normal and ccRCC patients in TCGA. Each column denotes a sample and each row represents the expression level of a gene. **c** Gene ontology (GO) enrichment analysis of 100 DEGs. The color scale represents *P* value ranging from red to yellow. The dot represents gene counts enriched in GO. **d** LASSO and SVM-RFE algorithms in the TCGA cohort

Gene expression dataset of TCGA, GSE66272, GSE16449 and GSE76351 were download from TCGA and GEO database. After quality detection and normalization of raw data, 2334, 981, 574 and 692 DEGs were respectively screened, and the results of Veen plot showed that 100 DEGs are intersected differentially expressed identified from four datasets (Fig. 1a). As expected, hierarchical clustering analysis of TCGA datasets was employed to exhibit the distribution of these genes, including 30 upregulated genes and 70 downregulated genes (Fig. 1b). To elucidate the function, gene ontology (GO) enrichment analysis was performed. Interesting, the results showed that these genes mainly were enriched in cellular inflammation, apoptosis and proliferation (Fig. 1c). We then used LASSO and the SVM-RFE algorithms to filter important DEGs for classifying normal and ccRCC patients. The results indicated that eight genes were correlative with patients’ survival (Fig. 1d).

### Integrated-signature mRNAs show clinical prognostic significance in ccRCC patients

**Fig. 2:**
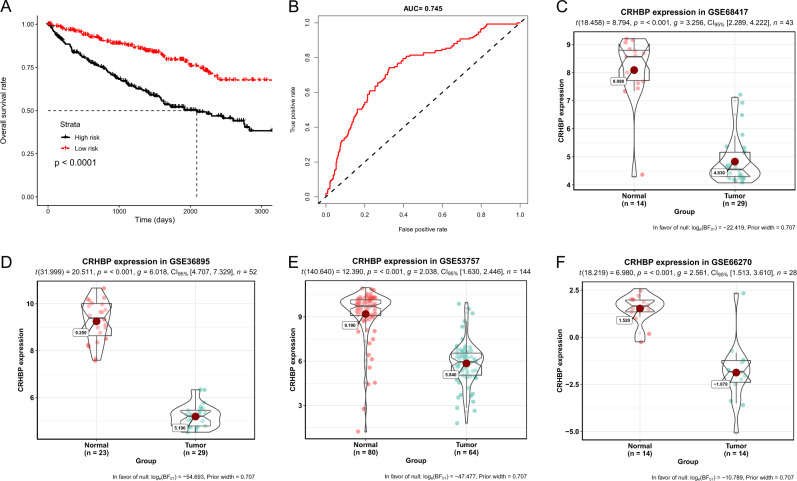
Three validated mRNAs may act as significant prognostic markers in ccRCC classification. **a** Kaplan–Meier analysis of overall survival in the light of patient median risk score to assess prognostic value. *P* values were calculated using the log-rank test. **b** A receiver operating characteristic (ROC) curve was analyzed by univariate and multivariate regression models based on patient risk scores. The score performance of ROC was validated by calculating the area under the ROC. **c**–**f** The mRNA expression of CRHBP between normal and ccRCC group in GSE68417, GSE36895, GSE53757, GSE66270

To investigate the correlation between eight genes and ccRCC patients, univariate and multivariate analyses were performed, and the results showed that three genes named *CRHBP, PSAT1, RAB25* were the most significant (*P* < 0.05) linked with patients clinicopathological characteristics (Table 1). Subsequently, a predictive model was conducted to determine whether those genes could serve as biomarkers [16]. Each patient was distributed a risk score in accordance with cox regression coefficients and divided into two groups via median risk score. The plots result of Kaplan–Meier showed that patients in high-risk group had shorter overall survival rate than those with low risk (*P* < 0.001, Fig. 2a), and the results of ROC indicated that these genes could be crucially important in ccRCC patients (Fig. 2b). Four independent microarray datasets were then validated in our bioinformatic analysis (Fig. 2c–f). The results of GSE68417, GSE36895, GSE53757 and GSE66270 datasets showed significantly lower CRHBP expression in ccRCC group than those in the adjacent non-cancer group, revealing that CRHBP could serve as biomarker for ccRCC prognosis and diagnosis.

**Table 1:** Univariate and multivariate analyses of clinicopathological characteristics and important genes with overall survival in TCGA KIRC cohort

	Univariate analysis		Multivariate analysis	
	HR (95% CI)	*P* value	HR (95% CI)	*P* value
TCGA KIRC set (*n* = 491)				
Age (>60 years vs ≤ 60 years)	**0.536 (0.385–0.744)**	**<0.001**	**0.594 (0.426–0.83)**	**0.0022**
Gender (male vs mfemale)	1.045 (0.749–1.458)	0.797		
Pathological stage(T1 + T2 vs T3 + T4)	**3.8 (2.706–5.335)**	**<0.001**	**2.509 (1.729–3.642)**	**<0.001**
Neoplasm grade (G1 + G2 vs G3 + G4)	2.716 (1.874–3.936)	<0.001	1.459 (0.972–2.19)	0.0682
ALDH6A1 (>median vs ≤ median)	0.384 (0.272–0.542)	<0.001	0.809 (0.532–1.231)	0.3228
CRHBP (>median vs ≤ median)	**0.437 (0.311–0.615)**	**<0.001**	**0.613 (0.425–0.885)**	**0.0089**
ESRRG (>median vs ≤ median)	0.516 (0.371–0.72)	<0.001	0.923 (0.633–1.344)	0.6743
FBP1 (>median vs ≤ median)	0.531 (0.382–0.737)	<0.001	0.796 (0.556–1.137)	0.2098
NNMT (>median vs ≤ median)	1.741 (1.253–2.42)	<0.001	1.066 (0.746–1.524)	0.7262
PSAT1 (>median vs ≤ median)	**2.653 (1.872–3.761)**	**<0.001**	**1.601 (1.089–2.353)**	**0.0166**
RAB25 (>median vs ≤ median)	**1.552 (1.121–2.149)**	**0.008**	**1.464 (1.047–2.047)**	**0.026**
SCARB1 (>median vs ≤ median)	0.747 (0.541–1.03)	0.075		

Bold values indicate statistical significance *P* < 0.05

### CRHBP is correlated with poor prognosis and significantly downregulated in ccRCC

**Fig. 3:**
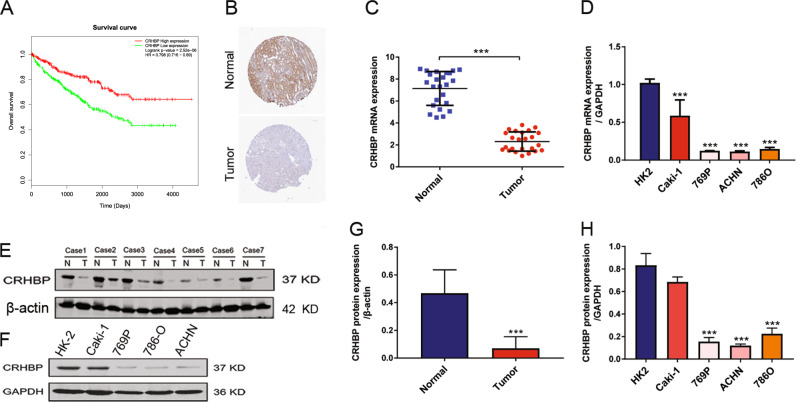
CRHBP expression is downregulated in ccRCC. **a** Survival curve of ccRCC patients with high and low CRHBP expression. **b** Representative immunohistochemistry (IHC) analysis of CRHBP between normal and tumor tissues from protein dataset (https://www.proteinatlas.org/). **c** Quantitative RT-PCR analysis of CRHBP expression in 24 pairs ccRCC tissues (right panel) and corresponding adjacent normal kidneys (left panel). **d** Quantitative RT-PCR analysis of CRHBP expression in human normal kidney cell line (HK-2) and ccRCC cell lines (Caki-1, 769P, ACHN, 786O). **e** Western blotting assay of CRHBP expression in seven pairs of human clinical ccRCC samples. β-actin was used as the loading control. **f** Western blotting assay of CRHBP expression in HK-2 and ccRCC cell lines. GAPDH was used as the loading control. **g**, **h** Representative quantitative analysis of CRHBP protein levels. Data are presented as the mean ± SD. ***P < 0.001 tumor tissue vs normal tissue. CcRCC cell lines vs HK-2 cell line. Statistical significance was performed with a two-tailed Student’s *t*-test

To verify the importance of CRHBP in ccRCC the survival analysis indicated that the ccRCC patients with high expression of CRHBP have longer survival overall than those with low levels of CRHBP (Fig. 3a). Subsequently, to further confirm the CRHBP expression levels in ccRCC tissues and cell lines, qRT-PCR and western blotting assay were performed to test the mRNA and protein levels. The results showed that both mRNA and protein expression of CRHBP in tumor tissues were significantly downregulated compared with adjacent non-tumor tissues (Fig. 3b, g). Similarly, the expression of CRHBP was significantly decreased in ccRCC cell lines of Caki-1, 769P, ACHN and 786O when compared with normal human kidney cell line (Fig. 3c, e, f, h). Simultaneously, results of immunohistochemistry (IHC) analysis according to dataset (https://www.proteinatlas.org/) of CRHBP also showed the same result (Fig. 3d), proving that CRHBP is a tumor suppressor gene and plays an important role in the progress in ccRCC.

### CRHBP suppresses cell migration and invasion of ccRCC cells

**Fig. 4:**
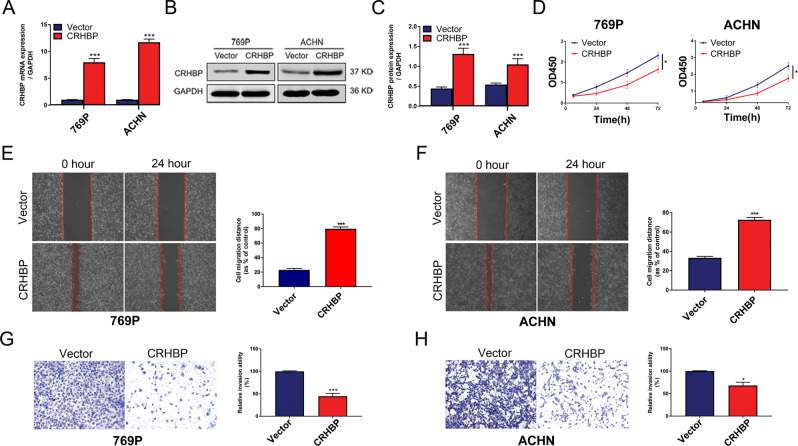
CRHBP expression level is related to migration and invasion of ccRCC cell lines. **a**, **b** CRHBP transfection efficiency was evaluated by RT-qPCR (**a**) and western blotting (**b**) in 769P and ACHN cell lines. **c** Representative quantitative analysis of CRHBP protein levels. **d** Cell Counting Kit-8 assay of ccRCC cells with stable overexpression of CRHBP vs vector at indicated time points. **e**, **f** Wound healing assay of stable overexpression of CRHBP vs vector in 769P and ACHN cells at 0 and 24 h. The cell migration distance (%) was calculated. Scale bar = 20 µm. **g**, **h** Transwell assay of 769P and ACHN cells with stable overexpression of CRHBP vs vector at 24 h. Relative invasion ability (%) was calculated. Scale bar = 20 µm. Data are presented as the mean ± SD. *P < 0.05, ***P < 0.001. CRHBP vs vector group

In order to explore the biological role of CRHBP in RCC progress, 769P and ACHN were respectively selected as in vitro cell models [17]. 769P and ACHN cells were infected by lentivirus against purinomycin to induce stable overexpression of CRHBP, and verified by qRT-PCR (Fig. 4a). Western blot analysis also confirmed the transfection efficiency in two cells (Fig. 4b, c). To test the proliferative ability of ccRCC cells, CCK-8 assay was employed in 769P and ACHN. As shown in Fig. 4d, the overexpression of CRHBP significantly inhibited proliferation of both 769P and ACHN in 72 h. Moreover, the results of wound healing assay indicated that the migration capacity of 769P and ACHN cells was significantly repressed in CRHBP-overexpressed group compared with that in vector group (Fig. 4e, f). Similarly, transwell assay showed that 769P and ACHN control cells acquired greater invasive capacity than corresponding CRHBP stable overexpression cells (Fig. 4g, h). These results implyed that CRHBP could oppress the capacity of proliferation, migration and invasion in ccRCC cells.

### CRHBP activates NF-κB signaling pathway in ccRCC cell lines

**Fig. 5:**
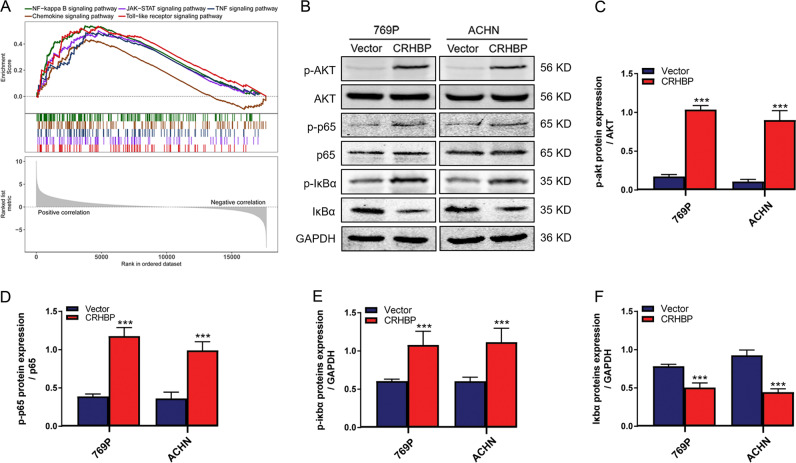
NF-κB signaling pathway is positively regulated by CRHBP in ccRCC cells. **a** Gene set enrichment analysis of TCGA clear cell renal cell carcinoma samples with low and high CRHBP mRNA expression. **b** Western blotting showed protein levels of phosphorylated-AKT, AKT, phosphorylated-p65, p65, phosphorylated-IkBα and IkBα. GAPDH was used as a loading control. **c**–**f** Quantification analysis of NF-kB signaling pathway correlative proteins. Data are presented as the mean ± SD. ***P < 0.001. CRHBP vs vector group

To investigate the potential mechanism of the cellular biological functions described above, GSEA was employed to discover the underlying signaling pathways in ccRCC progression according the median expression of CRHBP. Interestingly, multiple significant pathways were correlative with inflammation, and the top five significantly pathways were NF-κB, JAK-STAT, tumor necrosis factor, chemokine and toll-like receptor signaling pathways (Fig. 5a). To confirm whether the pathway of NF-κB was activated, phosphorylated-AKT (p-AKT), AKT, phosphorylated-p65 (p-p65), p65, phosphorylated-IkBα (p-IkBα) and IkBα were examined by western blotting (Fig. 5b). The result revealed that CRHBP group significantly increase the protein levels of p-AKT, p-p65 and p-IkBα, as well as decrease the expression of IkBα compared with vector group both in 769P and ACHN cells (Fig. 5c–f), indicating that CRHBP may be capable of promoting ccRCC inflammation in part via activating the NF-kB signaling pathway.

### CRHBP promotes ccRCC apoptosis through regulating p53-mediated mitochondria apoptotic pathway in vitro and inhibits tumor growth in vivo

**Fig. 6:**
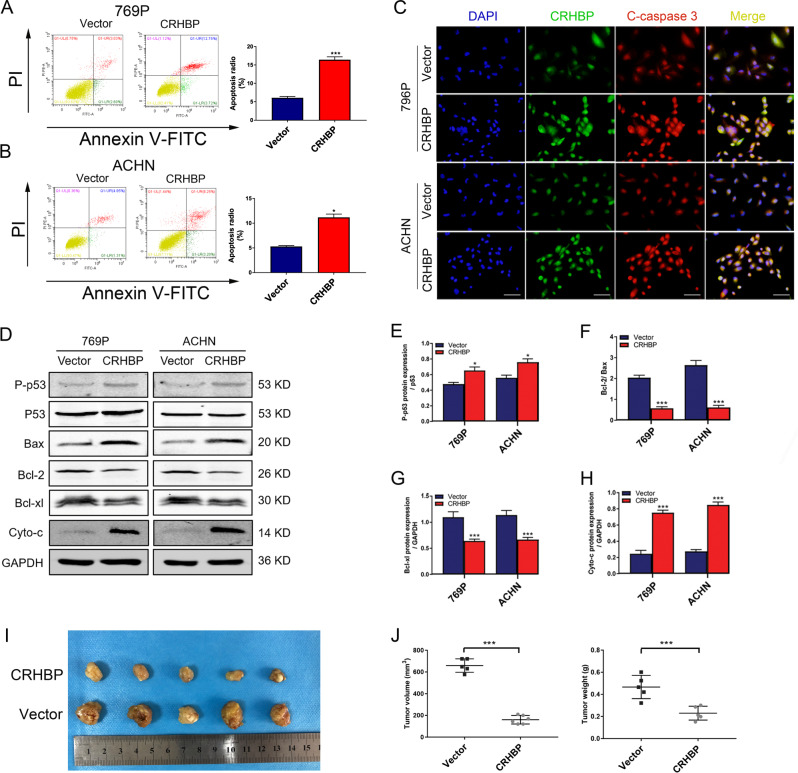
Overexpression of CRHBP induces ccRCC cellular apoptosis by targeting p53-mediated mitochondria apoptotic pathway. **a**, **b** Flow cytometric analysis was employed to detect apoptosis of 769P and ACHN cells. **c** Representative photomicrographs of immunofluorescence analysis of CRHBP and cleaved-caspase 3 from ccRCC cells. Scale bar = 20 µm. **d** Western blotting showed protein levels of phosphorylated-p53, P53, Bax, Bcl-2, Bcl-xl and cytochrome c. GAPDH was used as a loading control. **e**–**h** Quantification analysis of apoptotis-related proteins. Representative images of the isolated subcutaneous tumors in nude mice implanted with 769-P/Vector and 769-P/CRHBP cells (**i**). **j** Quantification analysis of tumor volume and weight between CRHBP overexpression and vector xenograft. Data are presented as the mean ± SD. *P < 0.05, ***P < 0.001. CRHBP vs vector group

Apoptosis is one of the most important events during the progress in ccRCC. To explore whether CRHBP induced ccRCC cellular apoptosis, we detected apoptosis of both 769P and ACHN cells by flow cytometry. The results showed that apoptotic cells of 769P and ACHN were significantly increased compared with vector cells especially in early apoptosis (annexin V + /PI, color = red) (Fig. 6a, b). Moreover, immunofluorescence analysis showed that the expression of cleaved-caspase 3 was markedly increased in ccRCC cells of CRHBP group compared with control cells (Fig. 6c). To further obtain insight into the possible mechanism by which CRHBP induced ccRCC cellular apoptosis, western blotting was employed to investigate apoptosis-related proteins (Fig. 6d). We found that CRHBP upregulated the expression of phosphorylated-p53 and cytochrome c whereas downregulated the ratio of Bcl-2/Bax and Bcl-xl compared with vector group (Fig. 6e–h). Collectively, these results suggest that CRHBP has a potential effect on promoting ccRCC cellular apoptosis through activating p53-mediated mitochondrial apoptosis pathway. To further explore the roles of CRHBP in cRCC in vivo, 769P cell with stable overexpression of CRHBP and control cell were subcutaneous injected into athymic nude mouse models, respectively. We discovered that CRHBP overexpression resulted in a significant reduction in tumor volume and weight compared with the control group tumors (Fig. 6i, j). According to our data, we demonstrated that CRHBP functions as an important suppressor and could negatively modulate tumorigenicity of ccRCC in vivo.

## Discussion

Accumulating evidence indicated that ccRCC is not sensitive to chemotherapy, radiotherapy as well as targeted therapy, resulting in recurrence rate and 5-year survival rate of the patient is still up to 40% and 20% after radical nephrectomy, respectively [18]. Therefore, management of ccRCC patient needs novel research on the mechanisms of tumorigenesis and metastasis to discover predictable and druggable biomarkers.

In the present study, we revealed that the expression of CRHBP is aberrantly decreased in ccRCC samples and cell lines, and positively associated with overall survival rate of patients. Besides, the levels of CRHBP are closely related to patients’ clinical stage and histologic grade, implying that it has important role in the pathogenesis of ccRCC. Then, lentivirus with overexpressed CRHBP was transfected into ccRCC cell lines to prove the results of bioinformatic analysis. Functionally, CRHBP could suppress the cellular capacity of proliferation, migration and invasion. In addition, it promoted inflammation and apoptosis in 769P and ACHN via regulating NF-κB and p53-related apoptotic pathways. Consistently, the data of tumor xenograft model indicated that overexpression of CRHBP inhibited tumor growth and metastasis in mice. All these results support a notion that CRHBP may be acted as a potential biomarker for ccRCC diagnosis and therapy.

CRHBP, a kind of circulating peripheral secreted protein expressed in hypothalamus cortical regions and pituitary gland, was reported to have effect on pregnancy, binge drinking as well as stress through binding corticotropin releasing factor [19–21]. Recently, other functions for CRHBP have been proposed in HCC and RCC [7, 8]. Xia et al. found low expression of CRHBP could predict a poor prognosis in HCC patients. Tezval et al. reported hypermethylation of CRHBP was correlative with its mRNA expression as well as clinicopathological parameters of RCC patients. However, all these researches have a limited clinical value because of including a small sample size. Conversely, our integrated analysis discovered the crucial gene according to two eminent public databases with more than 500 samples in ccRCC. Results indicated that decreased expression of CRHBP was found in ccRCC patients, revealing that CRHBP may be acted as an effective biomarker to diagnosis and prognosis in the clinical setting. Then, the bioinformatic result was validated and confirmed in independently datasets, clinical samples and ccRCC cell lines.

Up to now, accumulating evidence reported that CRHBP could attenuate the detriment of binge drinking, stress and pregnancy mainly through binding to its receptor such as corticotropin releasing factor type 2 and 3 [20–22]. CRHBP could also oppress proliferation of human endometrial adenocarcinoma cell line via activation of cAMP-PKA pathway [23]. But, the underlying mechanisms of CRHBP involved in the pathogenesis of ccRCC were still elusive. NF-κB pathway has been demonstrated to play an important role in RCC [24–26]. Tescalcin could promote RCC cells growth and metastasis by activating NF-κB pathway [24]. Besides, high expressions of p65 were correlated significantly with worse cancer-specific survival in ccRCC patients [25]. Amanda et al. also reported that knockdown of NF-κB1 could suppress the growth and invasion of RCC in vitro and in vivo [26]. Interestingly, our data are fully in agreement with the findings. GSEA results indicated that NF-κB pathway were altered in RCC grouped by CRHBP mRNA expression. Overexpression of CRHBP upregulated the protein expression of p-AKT, p-p65 and p-IkBα, while downregulated IkBα in vitro, suggesting that NF-κB pathway was inhibited by CRHBP. This may reveal that CRHBP functions of anti-inflammation might be through NF-κB pathway in ccRCC.

As we all know, inflammation is a vital trigger to the progression of cellular apoptosis [27]. The NF-κB hub subunit p65 translocates into nucleus to upregulate proinflammatory cytokines expression, leading to activation of apoptotic pathways [28]. It is well known that evasion of apoptosis is an important event during tumor cell growth and death. Many studies demonstrated that apoptosis played a crucial role in the treatment of neoplasm as it is a popular target of therapeutic strategies [29, 30]. Our apoptotic analysis indicated overexpression of CRHBP significantly increased the percentage of apoptotic ccRCC cells, suggesting CRHBP could induce ccRCC apoptosis. The mainly pathway in cell apoptosis is p53-mediated mitochondrial apoptosis pathway [31]. During cellular apoptosis, phosphorylation of p53 reduces interaction between p53 and facilitate bax transcript into mitochondria to induce the release of cytochrome c, initiating the cascade amplification effect of caspase family [32, 33]. These support the present data as the expressions of phosphorylated-p53, cytochrome c, bax, cleaved-caspase 3 were increased while the levels of bcl-2 and bcl-xl were decreased following the upregulation of CRHBP. Moreover, these observations are further corroborated in vivo assays. Together, these data are possible mechanisms of CRHBP may induce ccRCC cell apoptosis through activation of p53 mediated mitochondrial apoptosis pathway.

Together, present results demonstrate that CRHBP is a novel suppressor of ccRCC and further extend the notion that the underlying mechanisms of CRHBP anti-tumor capacity are associated with NF-κB pathway and p53-mediated mitochondrial apoptosis pathway. Nevertheless, there remain some limitations that are required to be solved. Whether there are two proteins or receptors that could interact with CRHBP to effect ccRCC progress because it is a secreted protein. Moreover, it is unclear how CRHBP regulates NF-κB signaling pathway and p53-mediated mitochondrial apoptosis pathway and whether it accepts the stimuli from environment and then still activates the pathways. In addition, CRHBP may act as a prognostic biomarker or potential target for ccRCC patient.

## References

[1] Capitanio U, Bensalah K, Bex A, Boorjian SA, Bray F, Coleman J (2019). Epidemiology of renal cell carcinoma. Eur Urol..

[2] Benjiamini Y, Hochberg Y (1995). Controlling the false discovery rate: a practical and powerful approach to multiple testing. J R Stat Soc B..

[3] Cairns P (2010). Renal cell carcinoma. Cancer Biomark.

[4] Capitanio U, Montorsi F (2016). Renal cancer. Lancet..

[5] Bhalla S, Chaudhary K, Kumar R, Sehgal M, Kaur H, Sharma S (2017). Gene expression-based biomarkers for discriminating early and late stage of clear cell renal cancer. Sci Rep..

[6] Zhao W, Zhao F, Yang K, Lu Y, Zhang Y, Wang W (2019). An immunophenotyping of renal clear cell carcinoma with characteristics and a potential therapeutic target for patients insensitive to immune checkpoint blockade. J Cell Biochem..

[7] Xia HB, Wang HJ, Fu LQ, Wang SB, Li L, Ru GQ (2018). Decreased CRHBP expression is predictive of poor prognosis in patients with hepatocellular carcinoma. Oncol Lett..

[8] Tezval H, Dubrowinskaja N, Peters I, Reese C, Serth K, Atschekzei F (2016). Tumor specific epigenetic silencing of corticotropin releasing hormone -binding protein in renal cell carcinoma: association of hypermethylation and metastasis. PLoS ONE..

[9] Gautier L, Cope L, Bolstad BM, Irizarry RA (2004). Affy-analysis of Affymetrix GeneChip data at the probe level. Bioinformatics..

[10] Robinson MD, McCarthy DJ, Smyth GK (2010). edgeR: a Bioconductor package for differential expression analysis of digital gene expression data. Bioinformatics..

[11] Tibshirani R (1996). Regression shrinkage and selection via the lasso. J R Stat Soc Ser B (Methodol)..

[12] Huang M-L, Hung Y-H, Lee W, Li R, Jiang B-R (2014). SVM-RFE based feature selection and Taguchi parameters optimization for multiclass SVM classifier. Sci World J..

[13] Qiu J, Peng B, Tang Y, Qian Y, Guo P, Li M (2017). CpG methylation signature predicts recurrence in early-stage hepatocellular carcinoma: results from a multicenter study. J Clin Oncol..

[14] Long J, Zhang L, Wan X, Lin J, Bai Y, Xu W (2018). A four-gene-based prognostic model predicts overall survival in patients with hepatocellular carcinoma. J Cell Mol Med..

[15] Yang K, Lu XF, Luo PC, Zhang J (2018). Identification of six potentially long noncoding RNAs as biomarkers involved competitive endogenous RNA in clear cell renal cell carcinoma. Biomed Res Int..

[16] Sun M, Song H, Wang S, Zhang C, Zheng L, Chen F (2017). Integrated analysis identifies microRNA-195 as a suppressor of Hippo-YAP pathway in colorectal cancer. J Hematol Oncol..

[17] Brodaczewska KK, Szczylik C, Fiedorowicz M, Porta C, Czarnecka AM (2016). Choosing the right cell line for renal cell cancer research. Mol Cancer..

[18] Lalani AA, McGregor BA, Albiges L, Choueiri TK, Motzer R, Powles T (2019). Systemic treatment of metastatic clear cell renal cell carcinoma in 2018: current paradigms, use of immunotherapy, and future directions. Eur Urol..

[19] Ketchesin KD, Stinnett GS, Seasholtz AF (2016). Binge drinking decreases corticotropin-releasing factor-binding protein expression in the medial prefrontal cortex of mice. Alcohol Clin Exp Res..

[20] Van Den Eede F, Van Broeckhoven C, Claes SJ (2005). Corticotropin-releasing factor-binding protein, stress and major depression. Ageing Res Rev..

[21] Petraglia F, Florio P, Simoncini T, Woods RJ, Giuntini A, Gremigni R (1997). Cord plasma corticotropin-releasing factor-binding protein (CRF-BP) in term and preterm labour. Placenta..

[22] Albrechet-Souza L, Hwa LS, Han X, Zhang EY, DeBold JF, Miczek KA (2015). Corticotropin releasing factor binding protein and CRF2 receptors in the ventral tegmental area: modulation of ethanol binge drinking in C57BL/6J mice. Alcohol Clin Exp Res..

[23] Graziani G, Tentori L, Portarena I, Barbarino M, Tringali G, Pozzoli G (2002). CRH inhibits cell growth of human endometrial adenocarcinoma cells via CRH-receptor 1-mediated activation of cAMP-PKA pathway. Endocrinology..

[24] Luo AJ, Tan J, He LY, Jiang XZ, Jiang ZQ, Zeng Q (2019). Suppression of Tescalcin inhibits growth and metastasis in renal cell carcinoma via downregulating NHE1 and NF-kB signaling. Exp Mol Pathol..

[25] Ng KL, Yap NY, Rajandram R, Small D, Pailoor J, Ong TA (2018). Nuclear factor-kappa B subunits and their prognostic cancer-specific survival value in renal cell carcinoma patients. Pathology..

[26] Ikegami A, Teixeira LFS, Braga MS, Dias M, Lopes EC, Bellini MH (2018). Knockdown of NF-kappaB1 by shRNA inhibits the growth of renal cell carcinoma in vitro and in vivo. Oncol Res..

[27] Lee HT, Gallos G, Nasr SH, Emala CW (2004). A1 adenosine receptor activation inhibits inflammation, necrosis, and apoptosis after renal ischemia-reperfusion injury in mice. J Am Soc Nephrol..

[28] Yang K, Li WF, Yu JF, Yi C, Huang WF (2017). Diosmetin protects against ischemia/reperfusion-induced acute kidney injury in mice. J Surg Res..

[29] Wong RS (2011). Apoptosis in cancer: from pathogenesis to treatment. J Exp Clin Cancer Res..

[30] Wang X, Wei S, Zhao Y, Shi C, Liu P, Zhang C (2017). Anti-proliferation of breast cancer cells with itraconazole: Hedgehog pathway inhibition induces apoptosis and autophagic cell death. Cancer Lett..

[31] Yuan S, Han Y, Ma M, Rao K, Wang Z, Yang R (2019). Aryl-phosphorus-containing flame retardants induce oxidative stress, the p53-dependent DNA damage response and mitochondrial impairment in A549cells. Environ Pollut..

[32] Fuchs Y, Steller H (2011). Programmed cell death in animal development and disease. Cell..

[33] Yin XM (2000). Signal transduction mediated by Bid, a pro-death Bcl-2 family proteins, connects the death receptor and mitochondria apoptosis pathways. Cell Res..

